# Effects of Seawater Intrusion on the Groundwater Quality of Multi-Layered Aquifers in Eastern Saudi Arabia

**DOI:** 10.3390/molecules28073173

**Published:** 2023-04-03

**Authors:** Mohammed Benaafi, S. I. Abba, Isam H. Aljundi

**Affiliations:** 1 Interdisciplinary Research Center for Membranes and Water Security, King Fahd University of Petroleum and Minerals, Dhahran 31261, Saudi Arabia; aljundi@kfupm.edu.sa; 2 Department of Chemical Engineering, King Fahd University of Petroleum and Minerals, Dhahran 31261, Saudi Arabia

**Keywords:** seawater intrusion, groundwater, irrigation hazards, water management, coastal regions

## Abstract

The degradation of groundwater (GW) quality due to seawater intrusion (SWI) is a major water security issue in water-scarce regions. This study aims to delineate the impact of SWI on the GW quality of a multilayered aquifer system in the eastern coastal region of Saudi Arabia. The physical and chemical properties of the GW were determined via field investigations and laboratory analyses. Irrigation indices (electrical conductivity (EC), potential salinity (PS), sodium adsorption ratio (SAR), Na%, Kelly’s ratio (KR), magnesium adsorption ratio (MAR), and permeability index (PI)) and a SWI index (*f_sea_*) were obtained to assess the suitability of GW for irrigation. K-mean clustering, correlation analysis, and principal component analysis (PCA) were used to determine the relationship between irrigation hazard indices and the degree of SWI. The tested GW samples were grouped into four clusters (C1, C2, C3, and C4), with average SWI degrees of 15%, 8%, 5%, and 2%, respectively. The results showed that the tested GW was unsuitable for irrigation due to salinity hazards. However, a noticeable increase in sodium and magnesium hazards was also observed. Moreover, increasing the degree of SWI (*f_sea_*) was associated with increasing salinity, sodium, and magnesium, with higher values observed in the GW samples in cluster C1, followed by clusters C2, C3, and C4. The correlation analysis and PCA results illustrated that the irrigation indices, including EC, PS, SAR, and MAR, were grouped with the SWI index (*f_sea_*), indicating the possibility of using them as primary irrigation indices to reflect the impact of SWI on GW quality in coastal regions. The results of this study will help guide decision-makers toward proper management practices for SWI mitigation and enhancing GW quality for irrigation.

## 1. Introduction

Groundwater (GW) is an important source of freshwater for plants and plays a significant role in meeting increased water demands for urban, industrial, and irrigation purposes in arid regions with low precipitation, high evaporation, and scarce surface water [1,2,3]. Owing to rapid population growth, urbanization, and industrialization, especially in coastal regions, water resources have become scarce and unsuitable for human use. The heavy abstraction of GW resources that exceed the natural recharge rate usually leads to hydrodynamic and hydrochemical misbalancing, thus triggering the inland movement of intruded seawater into coastal aquifers and elevating the salinity of coastal GW [4,5,6].

Notably, GW salinization poses a major danger to GW resources in coastal regions, reducing the quantity and quality of the water available for human and agricultural consumption. In coastal regions worldwide, GW resources are at risk of salinization owing to global warming and seawater intrusion (SWI) [7,8]. Rapid population growth, urbanization, industrialization, and agricultural development have all contributed to rising water demand [9,10,11,12,13]. As a result, seawater intrudes into coastal aquifers, changing GW salinity and quality to a level at which water becomes unsuitable for human consumption and agricultural use [14,15,16,17]. SWI is the main threat to GW in more than 501 coastal cities worldwide [10,18,19,20], and several studies have addressed SWI and GW quality in coastal regions [21,22,23,24]. Ko et al. (2021) [24] studied the impact of SWI on the GW quality in Htantabin Township, Myanmar. They found that GW was unsuitable for irrigation because of its elevated salinity and sodium and concluded that SWI was the main source of these elevated levels. Ding et al. (2020) [21] addressed the impact of SWI on GW quality in the Nile Delta, Egypt, and considered the salinity and alkalinity hazards of irrigated water and how they changed with distance from the sea. They found a landward decrease in GW salinity and alkalinity, with a significant relationship between EC, SAR, Na^+^, and Cl^−^ up to 15 km inland. They found that an increase in the mixing ratio of seawater was the main indicator of seaward SWI, with a maximum value at 5 km from the sea. They recorded a degradation in water quality due to SWI up to 15 km landward. However, the relationship between SWI and irrigation hazards (salinity, sodium, magnesium, and carbonate) was not addressed in their study. Sarkar et al. (2021) [22] investigated the GW quality of a coastal aquifer that was subjected to seawater conduction for irrigation in west Bengal, India. They found that 16% of the tested GW had high salinity hazards owing to increased seawater mixing by more than 1%. Their study also indicated the presence of silicate weathering, which controls the sodium concentration. Jahnke et al. (2019) [23] assessed the influence of SWI and desalinated brine effluent on GW quality in El Gouna, Egypt. They found that the coastal aquifer consisted of brackish water and intruded seawater and discussed the scaling issue of the mixing of GW and seawater due to the increased Ca^2+^ concentration in the solution caused by the ion exchange process. 

In this study, we developed a new approach to assess the impact of SWI on the quality of irrigation water using cluster analysis, irrigation hazard analysis, and SWI quantification. All irrigation hazards, including salinity, sodium, magnesium, and carbonate, were addressed and compared with SWI quantification indices to explore their impacts at different degrees of SWI. The aims of this research were to: (1) delineate the impact of SWI on the GW quality of a multi-layered aquifer system in the eastern coastal region of Saudi Arabia using K-mean cluster analysis, irrigation hazard indices, and a SWI quantification index (*f_sea_*). We believe that this is the first time that a combination of the saltwater intrusion index, cluster analysis, and irrigation risk indices has been used to study the effect of SWI on GW quality for irrigation in a multi-layer aquifer system. The results of this study may help decision-makers to sustainably manage GW resources and build an effective plan for managing SWI.

## 2. Study Area

**Figure 1:**
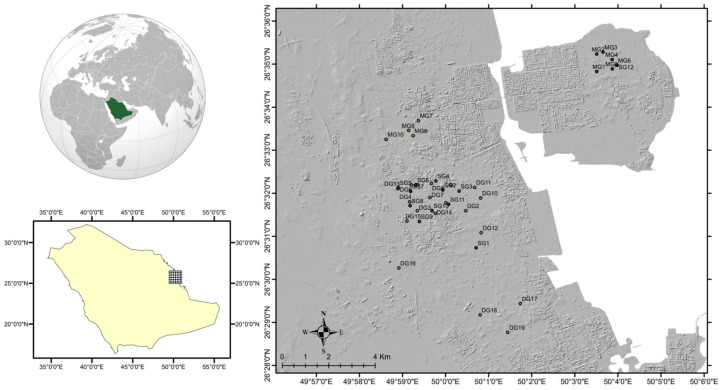
Map showing the location of the study area and GW samples.

The study area is located in the eastern region of Saudi Arabia along the western coast of the Arabian Gulf, within the administrative boundaries of the Al-Qatif governorate (Figure 1). It includes the coastal oases of Qatif and Tarout Island. The Qatif governorate is a coastal oasis with a dense population and urbanization and is bounded to the south by the Dammam governorate and to the north by the Al-Jubail governorate. It has a flat coastal plain, with surface elevations rising westward to a height of approximately 12 m. The study area consists of urban and farmland areas. However, in recent years, urban areas have encroached on croplands [13]. Water for irrigation on Qatif and Tarout Island is mostly collected from multilayered aquifers, including shallow, middle, and deep aquifers [25,26]. The higher extraction of GW was mainly from the middle and deep aquifers, owing to the salinization of the shallow GW [27]. The majority of the production wells in the study area tap through middle and deep aquifers and supply water for agriculture [27]. The shallow, middle, and deep aquifers were up to 30, 80, and 130 m below the ground surface, respectively. The water depth varied from <1 m near the shoreline to >5 m further inland.

## 3. Geological and Hydrogeological Setting

Table 1 depicts the geology of the research area, which consists of seven formations ranging from old to young: the Umm Er Redhuma, Rus, Dammam, Hadrukh, Dam, Hofuf, and Quaternary deposits. The Umm Er Radhuma Formation is a thick succession of carbonate rocks (limestone, dolomite, and dolomitic limestone) with an average thickness of 400 m [28,29,30]. The Rus Formation overlies the Emm Er Radhuma Formation and is divided into three parts with an average thickness of 10 m: lower, middle, and upper, comprising hard dolomitic limestone, chalky limestone, and microcrystalline limestone and shale, respectively [28,31]. The Dammam Formation stratigraphically overlies the Rus Formation. From bottom to top, it consists of five members: Midra, Saila, Alveolina, Khobar, and Alat Midra and Saila, which are made of marly shale with an average thickness of 10 m [32]. Alveolina is a microcrystalline limestone with a thickness ranging from 1 to 12 m [32]. The Khobar member of the Dammam Formation is mostly porous limestone, with an average thickness of 40 m [31,33]. The Alat member, composed of dolomitic marl and porous dolomitic limestone with a thickness ranging from 15 m in the outcrop area to roughly 70 m in the subsurface [32]. The Hadrukh Formation, which is strategically overlain by the Dammam Formation, is composed of interbedded sandstone layers with sandy marl and green shale. The thickness of the Hadrukh Formation ranges from a few meters to 90 m [28]. The Dam Formation formed above the Hadrukh Formation and is composed of clastic and microcrystalline limestone in the lower portion and massive reef facies limestone with voids in the top section [32]. The Dam Formation has an average thickness of 40 m in the outcrop and 90 m in the subsurface. The Hofuf Formation strata overlie the Dam Formation and are composed of conglomerates in the lower portion and sandy limestone in the upper part. Sabkha, Eolian sand, and marine terraces are examples of Quaternary deposits. They are found along the Arabian Gulf shore, with typical thicknesses ranging from 3 to 10 m [32].

The hydrogeological framework of the study area is a multi-layered aquifer system consisting of three principal aquifers separated by two aquitards. The three aquifers are the Neogene, Alat, and Al-Khobar. The Neogene aquifer is under unconfined conditions in the research area and is formed within the clastic unit of the Hadrukh Formation, with a transmissivity of 25.5–144 m^2^/h and a storativity of 0.01 [26,34]. The Alat aquifer is developed within the porous dolomitic limestone of the Alat Member of the Dammam Formation. The Hadrukh Shale Unit and Alat Shale aquitard separate the Alat aquifer from the overlying Neogene and underlying Khobar aquifers, respectively. The average depth of the Alat aquifer is 25 m, although it varies widely from a few meters at structural heights to more than 100 m near Ras Tanura. In eastern Saudi Arabia, the Alat aquifer has a transmissivity value between 1.16 and 310 m^2^/h. The storativity varied between 0.00013 and 0.00002. In the Qatif Oasis and Tarout Island (the research area), the transmissivity of the Alat aquifer was approximately 12.96 m^2^/h. The Khobar aquifer developed within the karstified and fissured dolomitic limestone of the Khobar member of the Dammam Formation. Both the upper and lower limits were bounded by the Alat shale aquitard and shale units (Midra and Saila, respectively) of the Dammam Formation. In both cities, Hassa and Qatif provided most of the water used for drinking and agricultural purposes. The hydraulic conditions of the Khobar aquifers in eastern Saudi Arabia range from 0.02–324 m^2^/h for transmissivity and 0.001–0.0001 for storativity. In the research area, the Khobar aquifer has a transmissivity of approximately 312 m^2^/h.

## 4. Proposed Methodology

**Figure 2:**
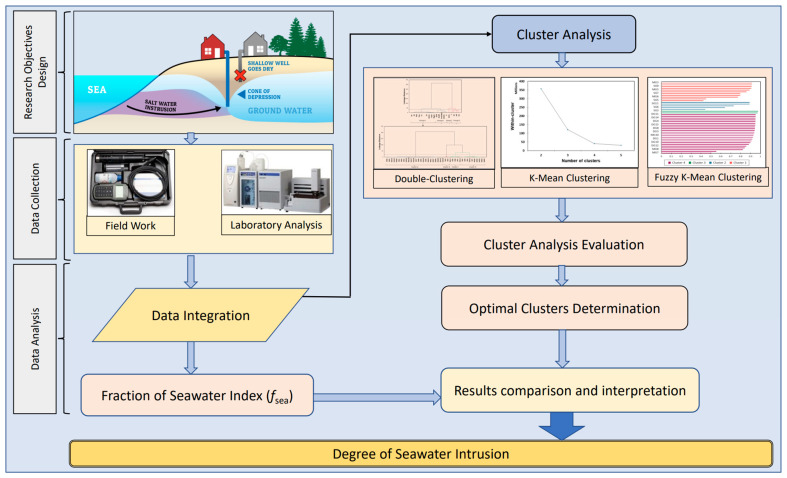
Flowchart showing the proposed methods and procedures.

Figure 2 shows a schematic representation of the research approach used in this study. A combination of fieldwork, laboratory analysis, clustering analysis, SWI quantification (fraction of seawater index (*f*_sea_)), and irrigation hazard analysis was utilized to determine the impact of the degree of SWI on the quality of the tested GW and its suitability for irrigation. The relationship between the SWI indicator (*f*_sea_) and the irrigation hazard indices—EC, PS, SAR, Na%, KR, MAR, and PI—was also evaluated using correlation and dimensional reduction statistical techniques (principal component analysis; PCA) to determine which group of indices would be most suitable for assessing GW quality in SWI-prone regions.

### 4.1. Field Investigation and Water Sampling

Forty-one wells in the eastern coastal region of Saudi Arabia that provide water for irrigation and human consumption were sampled in March 2021. Water samples were collected from observation and production wells. The observation wells tapped the shallow aquifer, which ranged in depth between 5–10 m. Production wells ranged in depth between 70–130 m, producing water from the middle and deep aquifers [13,25,26]. Water samples were taken from the wells after the water was pumped until the EC stabilized. Water sampling was performed in accordance with the guidelines of the US Environmental Protection Agency [35].

We utilized a portable Hanna GPS Multiparameter Meter (HI9829) to perform in situ measurements of pH, temperature, salinity, oxidation-reduction potential (ORP), EC, and dissolved oxygen (DO). Water samples were stored in polyethylene bottles before being filtered in real-time through membranes with a pore size of 0.45 μm. Two separate 250 mL sampling kits were prepared for anion, major cation, and trace element analyses using the filtered samples. Ultrapure HNO_3_ was used to acidify the samples for major cation and trace-element analyses.

### 4.2. Laboratory Analysis

Major cations (Na^+^, K^+^, Ca^2+^, and Mg^2+^) and major anions (Cl^−^, F^−^, Br^−^, SO_4_^2−^, and NO_3_^−^) were analyzed using high-performance ion chromatography (IC) at the King Fahd University of Petroleum and Minerals. Bicarbonate was measured using acid titration in the laboratory, and the level of total dissolved solids (TDS) was determined using gravimetric analysis. For quality control, blank, duplicate, and blind water samples were analyzed to verify the precision and calibration of the equipment. The ionic charge balance for all the water samples was cross-checked as follows:

(1) $$charge\;balance\left(\%\right)=\frac{\sum meq\;Cations-\sum meq\;Anions}{\sum meq\;Cations+\sum meq\;Anions}$$

The charge balance ranged from 0.3% to 5%, with an average of 3.5%. The charge balance for all GW samples was less than the 10% accepted criterion.

### 4.3. K-Mean Cluster Analysis

K-mean clustering is widely used as a data grouping method for large datasets and is one of the simplest non-hierarchical clustering and unsupervised learning algorithms [36]. K-means is a centroidal clustering method in which data are divided into subsets and assigned to clusters based on their centroids. The clusters aim to minimize the mean squared shortest distance (MSSD) between every point and its nearest cluster mean (centroid). The observations for each cluster exhibited similar properties and characteristics. K-mean clustering was performed in four steps [37]: (1) defining the number of clusters, (2) determining the centroid of each cluster, (3) quantifying the distance from each instance to the cluster centroid, and (4) allocating the instance to the closest centroid. The algorithms repeat these steps until there is no change in the cluster centroids. K-mean clustering defines the cluster centroids according to the following equation:

(2) $$\mu_{k}=\frac{1}{N_{k}}\sum_{q=1}^{N_{k}}x_{q}$$

where $\mu_{k}$, $N_{k}$, and $x_{q}$ stand for the new centroid, number of instances, and q-data in *k*-th cluster.

### 4.4. Silhouette Score

The silhouette score or silhouette coefficient has been reported by many researchers as a key index for validating clustering analysis and determining the optimal number of clusters [38,39,40]. The silhouette coefficient ranges in value from −1 to 1, with high values indicating high performance in clustering analysis. Negative values indicate that an instance does not belong to a cluster. The silhouette coefficient was calculated using the following equation:

(3) $$S\left(i\right)=\frac{b\left(i\right)-a(i)}{Max(a\left(i\right),b\left(i\right))}$$

where $b\left(i\right)$ represents the average dissimilarity of instance (i) to other data points within the same cluster, and a(i) represents the lowest average dissimilarity of instance (i) to any other cluster.

### 4.5. Fraction of Seawater Index (f_sea_)

The degree of SWI into the tested GW was quantified through the fraction of the seawater index (*f*_sea_), using the approach proposed by Appelo and Postma (2005) [41]. The concentration of chloride ions as a conservative ion was used as the basis for quantifying the fraction of the seawater index (*f*_sea_). The fraction of the seawater index (*f*_sea_), which represents the degree of salinization and SWI into the GW system, was calculated as follows:

(4) $$f_{sea}=\frac{C_{Cl,sam}-C_{Cl,f}}{C_{Cl,sw}-C_{Cl,f}}$$

where C_Cl,sam_, C_Cl,sw_, and C_Cl,f_ stand for chloride concentrations in the GW samples, seawater, and freshwater, respectively. The values of the fraction of seawater index (*f*_sea_) range from 0 to 1, where zero reflects freshwater and one represents seawater.

### 4.6. Irrigation Water Quality Assessment

Several water quality indices, such as EC, PS, SAR, Na%, KR, MAR, and PI, have been widely used to analyze the water quality and its degree of suitability for irrigation purposes in various situations [22,42,43,44,45,46,47,48,49]. These indices reflect several hazards, including salinity, sodium, magnesium, and carbonate in irrigated soils and crops. In this study, the SAR, Na%, and KR were used to assess the sodium hazards of the tested GW. In addition, salinity hazards were evaluated based on EC and PS. The magnesium hazard in the tested GW samples was evaluated by computing the MAR. The PI was used to assess the carbonate hazard of the irrigated water.

#### 4.6.1. Sodium Adsorption Ratio (SAR)

One of the most crucial indices for evaluating the sodium hazard of irrigated water is the SAR. The SAR indices reflect the relative concentrations of Na^+^, Ca^2+^, and Mg^2+^ ions. Water quality can be classified into four categories based on the SAR values: excellent (<10), good (10–20), permissible (20–40), and unsuitable (>40) [48,49]. The SAR index was computed as follows:

(5) $$\mathrm{SAR}=\frac{{Na}^{+}}{\sqrt{\frac{{Ca}^{2+}+{Mg}^{2+}}{2}}}$$

#### 4.6.2. Sodium Percentage (Na%)

Na% is another index used for the classification of irrigated water and for reporting Na hazards [48]. According to the Na% values, the irrigated water was classified as excellent (<20%), good (20–40%), permissible (40–60%), or unsuitable (>60%) [48,49]. The Na% index was calculated as follows:

(6) $$\mathrm{Na}\%=\frac{{Na}^{+}+K^{+}}{{Ca}^{2+}+{Mg}^{2+}+K^{+}+{Na}^{+}}\times 100$$

#### 4.6.3. Kelly’s Ratio (KR)

The KR serves as another criterion for evaluating the risk of sodium contamination in irrigation water. It is used to classify water used in irrigation as suitable (<1) or unsuitable (>1) [45,46,47]. KR was calculated as follows:

(7) $$\mathrm{KR}={Na}^{+}\frac{{Na}^{+}}{{Ca}^{2+}+{Mg}^{2+}}$$

#### 4.6.4. Potential Salinity (PS)

The PS serves as a criterion for evaluating the effect of salinity in irrigation water on soil salinity and fertility [44]. According to Doneen (1964) [44], the PS index expresses the risk of high salt concentrations due to Cl^−^ and SO_4_^2−^, which can increase the osmotic potential of a soil solution. Irrigated water was classified as suitable (<3 meq/L), moderate (3–5 meq/L), or unsuitable (>5 meq/L). PS was computed as follows:

(8) $$\mathrm{PS}={\mathrm{Cl}}^{-}+\frac{1}{2}{{\mathrm{SO}}_{4}}^{2-}$$

#### 4.6.5. Electrical Conductivity (EC)

EC serves as the main index for evaluating the effect of irrigation water salinity on soil salinity and fertility [42,43]. The EC was measured in the field using a portable water quality meter and reflected the degree of GW salinization. Based on the EC values and according to the United States’ Salinity Laboratory classification, irrigation water is classified into four categories: excellent (<250 μS/cm), good (250–750 μS/cm), permissible (750–2250 μS/cm), and unsuitable (>2250 μS/cm) [43].

#### 4.6.6. Magnesium Adsorption Ratio (MAR)

The MAR index is typically used to evaluate the effects of irrigation water on soil quality and crop productivity [48,50]. This reflects the Mg, as well as the Mg/Ca ratio in the irrigation water. Water with a MAR index of <50 was classified as suitable, while >50 was classified as unsuitable for irrigation. The MAR index is expressed by the following equation:

(9) $$\mathrm{MAR}=\frac{{Mg}^{2+}}{{Ca}^{2+}+{Mg}^{2+}}\times 100$$

#### 4.6.7. Permeability Index (PI)

The PI was used to evaluate the carbonate and alkalinity hazards of irrigation water to soil and crops [44]. This reflects the hazardous effects of carbonate and bicarbonate in irrigation water on the permeability and infiltration rate of the soil [51]. Based on the PI, the suitability of water for irrigation was classified into three classes: excellent (>75%), good (25–75%), and unsuitable (<25%) [44,51]. The PI was calculated as follows:

(10) $$\mathrm{PI}=\frac{{Na}^{+}+\sqrt{{{HCO}_{3}}^{-}}}{{Ca}^{2+}+{Mg}^{2+}+{Na}^{+}}\times 100$$

## 5. Results and Discussion

### 5.1. Groundwater Hydrochemistry and Water Type

**Figure 3:**
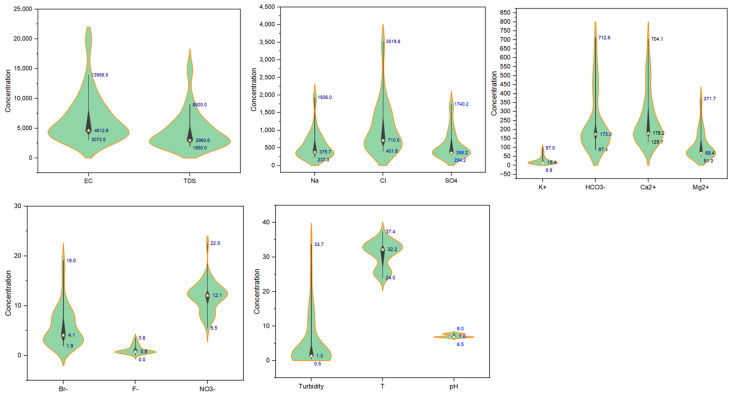
Violin plots showing the statistics and distribution of the physical and chemical parameters of the tested GW.

The minimum, maximum, and median values of major ions and physicochemical parameters in the GW of the multi-aquifer system in the eastern coastal region of Saudi Arabia are illustrated in Figure 3. Violin plots combine the best features of box plots and kernel density graphs, which helps researchers explore the data and provide summary statistics and density distributions of the tested variables [52]. The temperatures of GW samples range between 24–37.4 °C, reflecting different sampling depths. The pH of the GW ranged from slightly acidic (6.5) to slightly alkaline (8.0) and the concentration of dissolved oxygen varied from 1.01 to 5.7 mg/L. The EC and TDS varied widely, with values ranging between 3072–20,431 S/cm and 1955–15,560 mg/L, respectively. The minimum TDS value of the tested GW samples exceeded the permissible limit for drinking water (1000 mg/L) [53], indicating that they were unsuitable for drinking without treatment. The oxidation-reduction potential (Eh) and turbidity values of the tested GW ranged between −205–278 mV, and from 0.5–33.7 NTU, respectively. The water was composed of chloride and sulfate as the dominant anions with values ranging between 401.8–1064.1 mg/L (median: 710.5 mg/L) and 294.2–1740.2 mg/L (median: 385.2 mg/L), respectively. The predominant cations of the tested GW were sodium and calcium, with values ranging between 237.3–1936 mg/L (median: 375.7 mg/L) and 129.7–704.1 mg/L (median: 178.2 mg/L), respectively. Accordingly, the GW samples tested were Na and Na-Ca-Cl-SO_4_.

### 5.2. K-Mean Cluster Analysis

**Figure 4:**
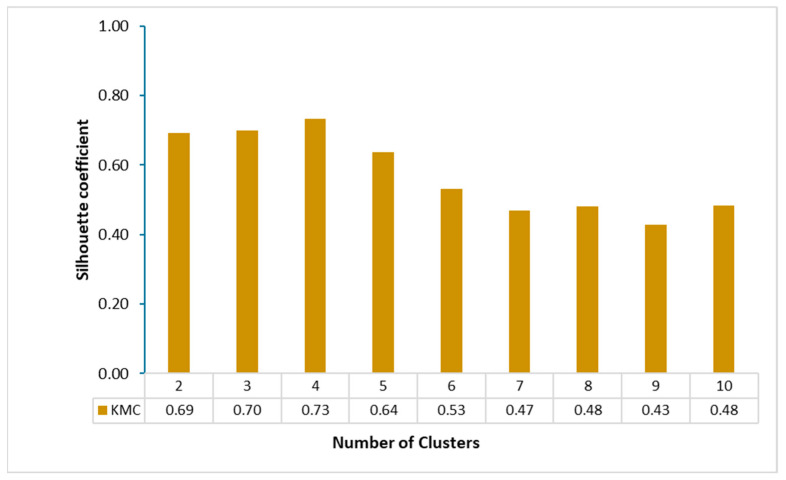
Plot showing number of clusters and silhouette scores of K-mean clustering.

**Figure 5:**
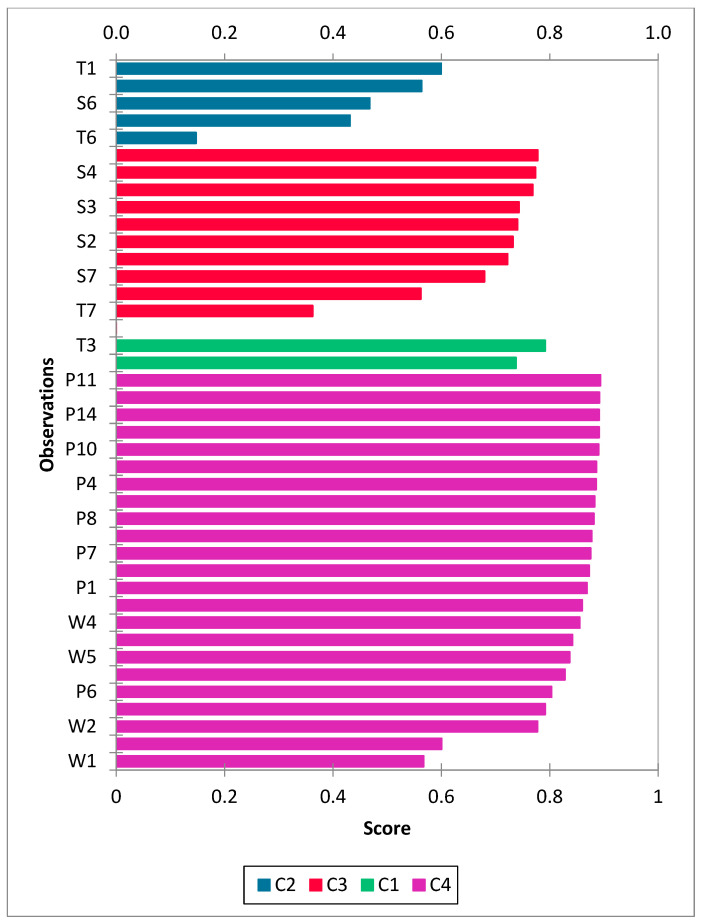
K-mean cluster plot showing silhouette scores for each observation.

K-mean clustering has been utilized by researchers in GW quality studies [40,54]. It is a useful tool for defining hydrochemical processes that control water quality for water quality assessment [55]. In this study, the K-mean clustering algorithm was applied to the entire GW dataset to group the water samples into different clusters. The optimal number of clusters was determined using the silhouette coefficient. As shown in Figure 4, the maximum silhouette coefficient was 0.73, corresponding to four clusters (C1, C2, C3, and C4). Clusters C1 and C2 were composed of 4.9% and 12.2% of the total GW samples, respectively, which were obtained mainly from a shallow aquifer with brackish and saline water. Cluster C3 contained GW samples from shallow and middle aquifers at 12.2% and 14.7%. Cluster C4 contained 56% of the total GW samples, mainly from deep aquifers. The silhouette coefficient of each sample is presented in Figure 5. The majority of water samples displayed silhouette values greater than 0.7, indicating that the samples were close to their clusters and more distant from other clusters.

### 5.3. Seawater Intrusion Quantification

**Figure 6:**
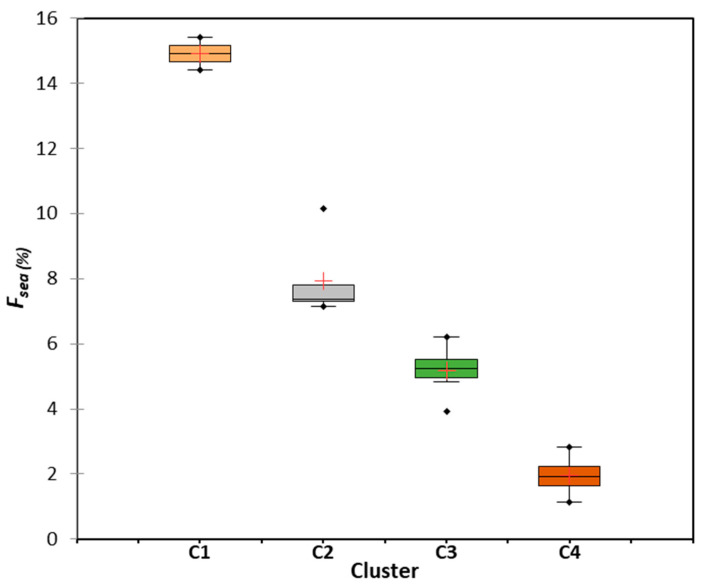
Box plots showing statistics values of the *f_sea_* for each cluster (C1, C2, C3, and C4) (+ represent the median values).

The degree of SWI into the GW sampled in the study area was measured using the seawater fraction (*f_sea_*) [22,41,56]. The results of the fraction of seawater index (*f_sea_*) for the GW samples tested in each cluster are illustrated in Figure 6. Additionally, the minimum, maximum, and mean values of *f_sea_* for each cluster (C1, C2, C3, and C4) were calculated, and are presented in Figure 6. Cluster C1 consisted of GW samples displaying a degree of SWI (*f_sea_*) ranging from 14.4% to 15.4%, with an average value of 14.5%. These samples were obtained from wells that only penetrated a shallow aquifer and were located within 2–3 km of the Arabian Gulf region. Cluster C2 comprises GW samples with values of *f_sea_* ranging from 7.2% to 10.1% and an average of 7.9%, indicating an intermediate degree of SWI. These samples were collected from a shallow aquifer and wells located 3–4 km from the Arabian Gulf. Cluster C3 consisted of GW samples with an *f_sea_* range of 3.9–6.2% and an average of 5.2%, revealing a low impact of SWI on GW quality. GW samples in cluster C3 were from shallow and middle aquifers within 3–4.7 km and 1–2 km of the Arabian Gulf, respectively. Cluster C4 consisted of GW samples with *f_sea_* ranging from 1.2–2.8% and an average value of 1.9%. The GW samples in cluster C4 were obtained from deep wells within 2–5 km of the Arabian Gulf (source of seawater). GW samples in C4 were from a deep aquifer, where SWI had very low or almost no impact on GW quality. Thus, SWI has not yet contaminated the deep layers of the multilayered aquifer system in the study area. Generally, the degree of SWI (*f_sea_*) was severe, intermediate, low, and very low in the GW samples in clusters C1, C2, C3, and C4, respectively.

### 5.4. Water Suitability for Irrigation

#### 5.4.1. Salinity Hazard

**Figure 7:**
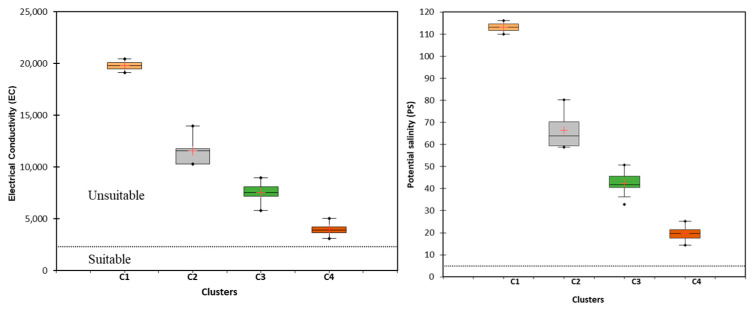
Plot depicting the variation in EC and PS indices of GW for the four clusters (C1, C2, C3, and C4) (+ represent the median values).

The salinity hazard of the GW was quantified using EC and PS. The salinity of irrigated water increases the osmotic pressure of soil solutions, hindering the absorption of water and nutrients by plants [57,58]. GW samples were analyzed for EC and PS to determine the extent to which they pose salinity risks. Figure 7 shows that the median EC values for all the examined GW classes were above 2250 S/cm, making them unsuitable for irrigation, according to the salinity classification scheme [43]. The GW samples in clusters C1, C2, C3, and C4 display EC values between 19,141.3–20,431.8 μS/cm, 10,315.9–13,958.5 μS/cm, 5809.6–8973.4 μS/cm, and 3072.5–5060.3 μS/cm, respectively. Salinity hazard variation was illustrated by comparing the median EC values of each cluster: C1, C2, C3, and C4 had median EC values of 19,786.5 S/cm, 11,591.9 S/cm, 7525.8, and 3993.3 S/cm, respectively. According to these findings, C1 contained the most hazardous GW, owing to its increased salinity, followed by C2, C3, and finally, C4. Despite this, the C4 GW samples were the least risky. Figure 7 shows the PS values for each GW sample cluster. Clusters C1, C2, C3, and C4 had PS values between 110–116 meq/L, 59–80 meq/L, 33–51 meq/L, and 14–25 meq/L, respectively. According to Kelley, 1951 [45], all GW samples had PS values greater than the acceptable threshold of 5 meq/L, with median values of 113 meq/L, 67 meq/L, 42 meq/L, and 20 meq/L for C1, C2, C3, and C4, respectively. Therefore, the high salinity hazards of GW in C1 correspond to a high degree of SWI (Figure 4). Additionally, the degree of salinity hazards (EC and PS) in the tested GW decreased as the degree of SWI decreased (Figure 4). These results are consistent with those of previous studies conducted in the coastal regions of India and Egypt [21,22]. Both studies found that the salinity hazards of irrigation water increased in conjunction with the extent of seawater incursion into the region.

#### 5.4.2. Sodium Hazard

**Figure 8:**
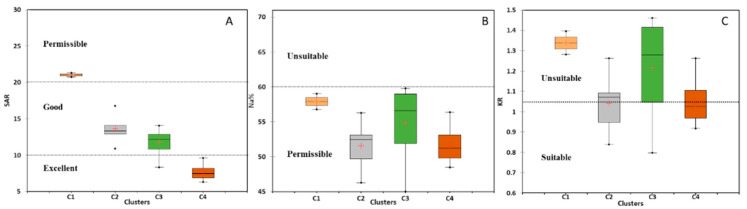
Variations in the sodium irrigation hazards of tested GW for all clusters (C1, C2, C3, and C4) (+ represent the median values). (**A**) SAR, (**B**) Na%, and (**C**) KR.

Scholars usually evaluate the sodium hazards of irrigated water using three indices: SAR, Na%, and KR [42,59,60]. In the current study, these three irrigation indices were applied to the GW samples to assess the degree of Na hazard. As illustrated in Figure 8A, the GW samples in clusters C1, C2, C3, and C4 display SAR values that range from 20.8–21.3, 10.9–16.8, 8.3–14.0, and 6.3–9.6, respectively. Based on these results and the USSL (United States Salinity Laboratory) classification scheme [43], the tested GW samples were placed in a suitable category for irrigation. However, GW samples from C1 and C4 were within the permissible and excellent classes, respectively. Clusters C2 and C3 contained water samples that fell within the good water quality category. The variation in the degree of Na hazards among the four clusters of GW samples was illustrated by comparing the median SAR value of each cluster. Clusters C1, C2, C3, and C4 had median SAR values of 21.0, 13.6, 11.8, and 7.5, respectively. The variation in the median SAR values between the four clusters indicated that the GW of C1 displayed the highest Na hazards, followed by C2, C3, and C4. This variation in SAR was compatible with the variation in the degree of SWI (*f_sea_*), with a greater influence on GW samples in C1, followed by C2, C3, and C4. Therefore, the SAR index may have more weight in assessing the water quality for irrigation in regions affected by SWI. Figure 8B depicts the results of the Na% index and shows that, according to the water quality guidelines, [46,61] all tested GW samples are within the permissible category. The Na% values in C1, C2, C3, and C4 range between 56.8–59.0, 46.3–56.3, 45.0–59.8, and 48.5–56.4, respectively. The median Na% values of the four clusters were 57.9, 52.5, 56.6, and 51.2, respectively, displaying less variation among the four groups, with the maximum values in C1, followed by C3, C2, and C4. The KR values of water samples from the four clusters are illustrated in Figure 8C. It is clearly shown that, with values of 100%, 50%, 75%, and 50% of the water samples in clusters C1, C2, C3, and C4, respectively, they were unsuitable for irrigation purposes and may reflect Na hazards. However, 50%, 25%, and 50% of the water samples in C2, C3, and C4, respectively, were within the suitable classes for irrigation [46,61]. The median KR values varied among clusters, with C1, C2, C3, and C4 with values of 1.3, 1.1, 1.3, and 1.0, respectively. Although this trend in KR variation was consistent with Na%, it was incompatible with SAR and the SWI index (*f_sea_)*. In general, the SAR index is the only Na hazard index that showed a trend of compatibility with the SWI index, indicating the higher reliability of this index for assessing water quality in coastal regions affected by SWI. Increased SAR concentrations with increased landward SWI have also been reported in the Nile Delta, Egypt [21]. As a result, the findings of the current study regarding the variation in SAR index and SWI are consistent with those of earlier research. However, this is the first study to highlight the significance of the SAR index among other Na hazard indices as the most helpful tool for assessing Na hazards in GW in coastal regions with varying degrees of SWI.

#### 5.4.3. Magnesium Hazard

**Figure 9:**
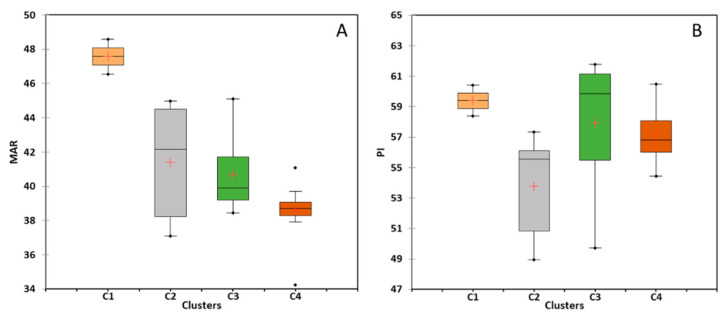
Plot showing the variation of the (**A**) MAR index and (**B**) PI of the tested GW. (C1, C2, C3, and C4) (+ represent the median values).

The MAR was used to assess the toxicity of GW to magnesium [48,56,62]. The MAR index findings for the four GW clusters are shown in Figure 9A, indicating significant heterogeneity between the clusters. The MAR values range between 37.1–46.6, 34.2–38.4, 45.0–48.6, and 41.1–45.1, respectively, for GW samples in clusters C1, C2, C3, and C4. According to these results, all GW samples fell into the suitable category (MAR < 50), which is appropriate for irrigation purposes [48]. However, the median MAR values varied between clusters, with values of 47.6, 42.1, 39.9, and 38.7 for C1, C2, C3, and C4, respectively. This variation has an increasing trend in clusters C4, C3, C2, and C1, which is compatible with the variation in the degree of SWI (*f_sea_*) (see Figure 4). Accordingly, the MAR index can contribute significantly to water quality assessment in coastal regions affected by SWI. The findings of this study are consistent with those of previous studies. For instance, Sarkar et al., (2021) [22] found that the GW in the coastal aquifer of Bengal, influenced by SWI, showed increased magnesium hazards. Similar research by Sangadi et al., (2022) [56] revealed increasing Mg^2+^ risks in brackish GW in coastal Andhra Pradesh, India.

#### 5.4.4. Carbonate Hazard

The PI is used to assess the detrimental effects of GW carbonates on the structure and permeability of the soil [44]. Two examples of these negative effects are the corrosion of irrigation water pipelines caused by elevated bicarbonate levels and the precipitation of Ca^2+^ and Mg^2+^ as calcite and magnesite, respectively [63]. Although no carbonate was detected in any of the water samples used in this study, bicarbonate was confirmed to be the only source of total alkalinity. Figure 9B shows the PI values of the four GW clusters. According to the stated recommendations by Doneen, 1964 [44], (namely 25–75%), all of the GW samples that were analyzed fell within a range of 49.0–61.8% and were considered good and suitable for irrigation [48,63]. Consequently, there was no considerable carbonate risk associated with the use of the tested GW samples for irrigation.

Additionally, the median PI values for GW in clusters C1, C2, C3, and C4 were 59.4, 55.6, 59.9, and 56.8, respectively. Comparing these results with the SWI results (see Figure 4), we found no compatibility in the pattern of variation, indicating that SWI had no impact on the carbonate hazards of the tested GW. Some studies have discussed the carbonate risks in coastal regions under SWI conditions. For example, according to Sangadi et al., (2022) [56], brackish GW is suitable for irrigation in the eastern coastal region of India. Similarly, Sarkar et al., (2021) [22] found that GW under SWI in West Bengal, India, is suitable for irrigation.

### 5.5. Relationship between Seawater Intrusion and Irrigation Hazards

**Figure 10:**
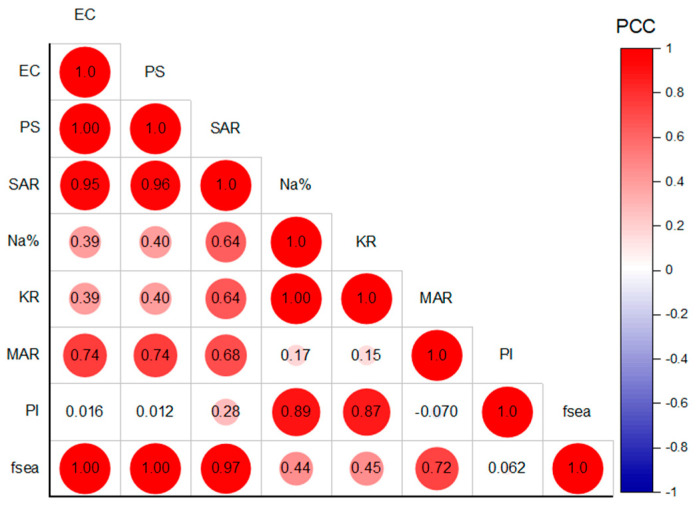
Correlation map of the SWI index (*f*_sea_) and irrigation hazard indices (EC, PS, SAR, Na%, KR, MAR, and PI) (CC is the Pearson correlation coefficient).

**Figure 11:**
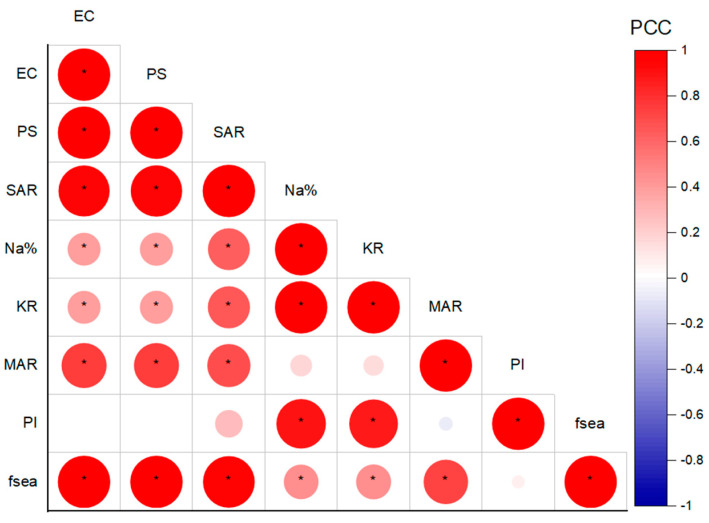
Correlation map of *f*_sea_, EC, PS, SAR, Na%, KR, MAR, and PI, with the significance mark (*p*-value <= 0.05 represented by *).

The impact of SWI on GW quality for irrigation was determined through multivariate statistical analyses, including correlation and PCA. Pearson correlation analysis was performed on the irrigation hazard index (EC, PS, SAR, Na%, KR, MAR, and PI) and SWI index (*f_sea_*) datasets to unravel the associated relationship. As shown in Figure 10 and Figure 11, strong and significant (*p*-value < 0.05) correlations were observed between *f_sea_* and the EC, PS, and SAR indices (high intensity of red color in Figure 10), with a correlation coefficient (PCC) greater than 0.97, indicating the severe impact of SWI on the salinity and sodium hazards of irrigated water. In addition, there was a moderate correction between the *f_sea_* and MAR indices, with a coefficient of correlation (PCC) of 0.72, revealing a moderate impact of SWI on the magnesium concentration and the hazards of the tested GW. A low correlation was observed between the *f_sea_* and Na% and KR indices, with a correlation coefficient of 0.44 and 0.45, respectively. This indicates that these indices may not be suitable for representing sodium hazards in regions affected by SWI. No correlation was observed between *f_sea_* and PI, revealing that SWI did not contribute to increasing the carbonate hazards of the tested GW. The results of the correlation between irrigation hazards are presented in Figure 10. A strong correlation is observed between the EC, PS, and SAR, with a correlation coefficient greater than 0.95, revealing a more significant relationship and the source of the hazards. In addition, the MAR index showed a moderate relationship with the EC, PS, and SAR indices, with a correlation coefficient range of 0.68–0.74. Generally, the correlation results show a close relationship between the salinity hazard indices (EC and PS), the sodium hazard index (SAR), and the magnesium hazard index (MAR). Thus, these irrigation hazard indices are more reliable for assessing GW quality in coastal regions affected by SWI.

**Figure 12:**
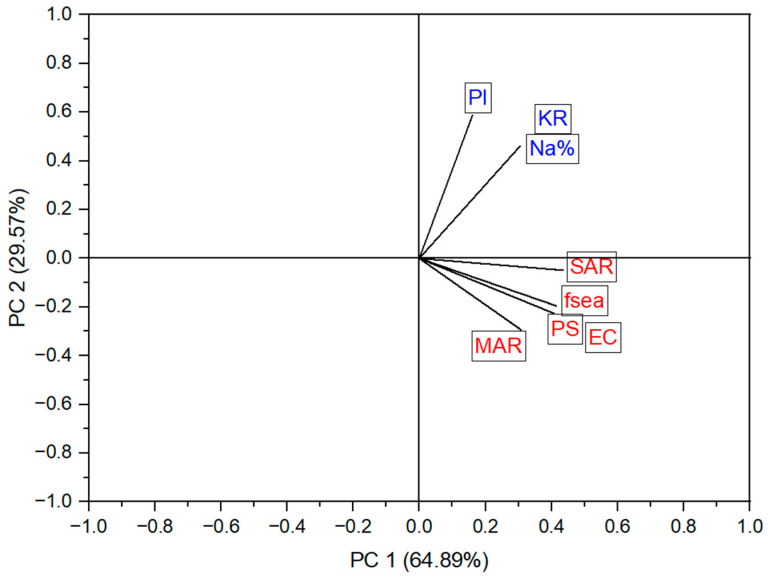
Two-dimensional plot of the principal component factors (PC1 and PC2).

PCA was performed in order to evaluate the relationship between the irrigation hazard indices and SWI (*f_sea_*), to define index groups that can effectively assess GW quality for irrigation in coastal regions, and to determine the degree of SWI impact on irrigated GW and its hazardous levels. Table 2 and Table 3 and Figure 12 show the results of applying PCA to the dataset of irrigation hazards and SWI indices for the 41 observations (water samples). The PCA revealed two groups of observed indices related to the two principal factors (PC1 and PC2). The first group of indices included EC, PS, SAR, MAR, and *f_sea_*, which correlated significantly with PC1 with correlation coefficients of 0.93, 0.93, 0.99, 0.70, and 0.94, respectively (Table 2). Table 3 illustrates the contribution of each index to PC1 and PC2. EC, PS, SAR, MAR, and *f_sea_* significantly contributed to the construction of PC1, with a percentage range of 16.5–18.9%. The irrigation hazards (EC, PS, SAR, and MAR) in PC1 were grouped with the SWI index, *f_sea_*, reflecting the inverse impact of SWI on GW quality for irrigation. Thus, PC1 can be interpreted as a factor that primarily describes the salinity, sodium, and magnesium hazard variations in the GW of the study area. The second group of indices consisted of Na%, KR, and PI, which were mainly correlated with PC2 with correlation coefficients of 0.71, 0.71, and 0.90, respectively. These indices contributed to constructing the PC2, with a total percentage of 77.1%. Therefore, PC2 can be used to assess carbonate hazards in the tested GW. According to the results of the PCA analysis, the first group of irrigation hazard indices (EC, PS, SAR, and MAR) coupled with the SWI index *f_sea_* can be used effectively to construct a water quality index for GW quality assessment in the region affected by SWI.

## 6. Conclusions and Recommendations

In the current study, we utilized integrated clustering, irrigation index analysis, and the seawater intrusion (SWI) index (*f_sea_*) to assess the impact of SWI on groundwater (GW) resources in the eastern coastal aquifers of Saudi Arabia. The tested GW was classified into four main clusters, namely C1, C2, C3, and C4, representing different degrees of SWI from high to low. The results of the irrigation hazard indices, including EC, PS, SAR, Na%, KR, and MAR, revealed that GW is unsuitable for irrigation owing to salinity hazards. In contrast, GW displayed suitable categories for sodium, magnesium, and carbonate hazards. However, an increasing trend in salinity, sodium, and magnesium hazards was associated with an increasing degree of SWI, in ascending order, in clusters C4, C3, C2, and C1.

The irrigation hazard indices were statistically correlated with the SWI index (*f_sea_*) to understand the influence of SWI on GW quality. Salinity (EC and PS) and sodium hazards showed a significant increase due to SWI; however, a moderate increase in magnesium hazards (MAR) was observed. In contrast, no carbonate hazards (PI) are associated with SWI.

The PCA resulted in two groups of irrigation indices. The main groups were the EC, PS, SAR, and MAR indices. They are associated with the SWI index *f_sea_* reflecting the possibility of using these indices to assess GW quality for irrigation in regions with SWI. Decision makers can utilize such associations between irrigation hazards and SWI indices as effective indicators to monitor water quality and implement proper management practices. It is recommended that the results of the current study be implemented in order to generalize irrigation water quality indices that consider increased weighting for the irrigation indices (EC, PS, SAR, and MAR) to be applied in water quality assessments in coastal regions.

## Figures and Tables

**Table 1:** Lithological rock units and associated hydrogeological units in the eastern coastal region of Saudi Arabia.

Formation	Member	Lithology	Description	Thickness (m)	Hydrogeologic Unit
Quaternary Deposits		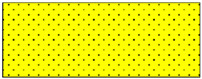	Eolian Sands and Sabkha	0–10	
Hofuf		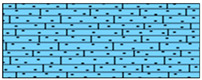	Sandy Limestone	0–95	Neogene Aquifer
Dam		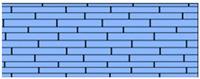	Massive Limestone	0–90	
		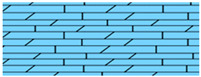	Dolomitic Limestone		
Hadrukh		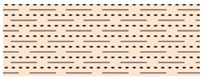	Sand and Shale	0–90	
		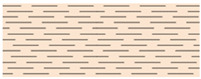	Shale		
		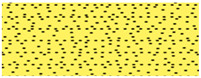	Sandstone		
		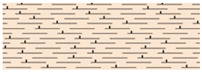	Marl		Marl Aquitard
Dammam Dammam	Alat	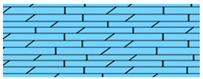	Dolomitic Limestone	0–80	Alat Aquifer
		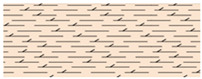	Dolomitic Shale	0–30	Alat Aquitard
	Khobar	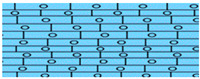	Limestone	0–70	Khobar Aquifer
	Alevolina	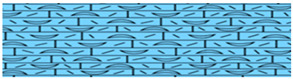	Fossiliferous Limestone	0–20	Limestone Aquitard
	Saila	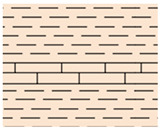	Shale with limestone	0–10	Shale Aquitard
	Midra				
Rus		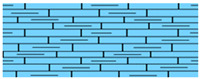	Marl and chalky limestone	60–100	Shale Aquitard
Umm Er Radhuma		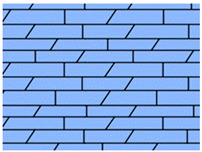	Dolomitic Limestone	200–400	UER Aquifer

**Table 2:** Factor loading of the principal component analysis.

	EC	PS	SAR	Na%	KR	MAR	PI	*f_sea_*
F1 (64.89%)	0.93	0.93	0.99	0.70	0.70	0.70	0.37	0.94
F2 (29.57%)	−0.35	−0.35	−0.08	0.71	0.71	−0.45	0.90	−0.30

**Table 3:** Percentage of indices contribution factor loadings.

	EC	PS	SAR	Na%	KR	MAR	PI	*f_sea_*
F1 (64.89%)	16.5	16.6	18.9	9.4	9.3	9.4	2.6	17.2
F2 (29.57%)	5.2	5.1	0.2	21.4	21.2	8.6	34.5	3.8

## Data Availability

Data will be available on request from the corresponding authors.

## Sample Availability

Samples of the compounds are available from the authors.
